# Evaluation of the Response of HNSCC Cell Lines to γ-Rays and ^12^C Ions: Can Radioresistant Tumors Be Identified and Selected for ^12^C Ion Radiotherapy?

**DOI:** 10.3389/fonc.2022.812961

**Published:** 2022-02-25

**Authors:** Lianghao Ding, Brock J. Sishc, Elizabeth Polsdofer, John S. Yordy, Angelica Facoetti, Mario Ciocca, Debabrata Saha, Arnold Pompos, Anthony J. Davis, Michael D. Story

**Affiliations:** ^1^ Univeristy of Texas Southwestern Medical Center, Department of Radiation Oncology, Dallas, TX, United States; ^2^ Medical Physics Unit & Research Department, Foundazione Centro Nazionale di Adroterapia Oncologica (CNAO), Pavia, Italy

**Keywords:** carbon ion radiotherapy, head and neck squamous cell carcinoma, radioresistance, relative biological effectiveness, prediction of radioresponse

## Abstract

Head and neck squamous cell carcinoma (HNSCC) is the sixth most common malignancy worldwide. Thirty percent of patients will experience locoregional recurrence for which median survival is less than 1 year. Factors contributing to treatment failure include inherent resistance to X-rays and chemotherapy, hypoxia, epithelial to mesenchymal transition, and immune suppression. The unique properties of ^12^C radiotherapy including enhanced cell killing, a decreased oxygen enhancement ratio, generation of complex DNA damage, and the potential to overcome immune suppression make its application well suited to the treatment of HNSCC. We examined the ^12^C radioresponse of five HNSCC cell lines, whose surviving fraction at 3.5 Gy ranged from average to resistant when compared with a larger panel of 38 cell lines to determine if ^12^C irradiation can overcome X-ray radioresistance and to identify biomarkers predictive of ^12^C radioresponse. Cells were irradiated with ^12^C using a SOBP with an average LET of 80 keV/μm (CNAO: Pavia, Italy). RBE values varied depending upon endpoint used. A 37 gene signature was able to place cells in their respective radiosensitivity cohort with an accuracy of 86%. Radioresistant cells were characterized by an enrichment of genes associated with radioresistance and survival mechanisms including but not limited to G2/M Checkpoint MTORC1, HIF1α, and PI3K/AKT/MTOR signaling. These data were used in conjunction with an *in silico*-based modeling approach to evaluate tumor control probability after ^12^C irradiation that compared clinically used treatment schedules with fixed RBE values vs. the RBEs determined for each cell line. Based on the above analysis, we present the framework of a strategy to utilize biological markers to predict which HNSCC patients would benefit the most from ^12^C radiotherapy.

## Introduction

The potential therapeutic advantage of particle radiotherapy was recognized in the 1940s and was based upon the physical properties of the energy deposition patterns of said particles. Since that time, particle therapy has continuously developed predominantly based upon advances in engineering, imaging, and physics. The first dedicated clinical heavy ion therapy center was opened in 1994, and this facility focused on the use of accelerated carbon ions because of the physical and biological advantages over photons and protons including steeper lateral dose penumbra at greater depths in the body, a higher LET which results in a higher relative biological effectiveness (RBE) and took into account the experience gained at the Lawrence Berkeley Laboratory where the initial results for the use of heavy charged particles as a cancer therapy took place (1–4).

Since then, carbon ion radiotherapy (CIRT) has been used against intracranial cancers, head and neck cancers, primary and metastatic lung cancers, gastrointestinal tumors, sarcomas, prostate cancer, breast cancer, and pediatric cancers at what has grown to become 12 carbon centers across the globe, although none currently exist in the USA. CIRT has been shown to exert a strong antitumor effect in tumors resistant to conventional photon therapy; however, some tumor sites have been less amenable to therapy over concerns for the response of adjacent normal tissues. Whereas, the efficacy of CIRT has been shown for nonsquamous tumors like mucosal melanomas (5, 6), adenocarcinomas and sarcoma (7, 8), and adenoid cystic carcinomas (9, 10); in tumors that are considered radioresistant or chemoresistant, the use of CIRT for squamous cell carcinomas, the most common type of head and neck cancer, has been limited (11–13).

Given the technologic advances in recent years for proton and heavy ion radiotherapy, the potential benefit from the increased conformity of charged particles and higher LETs seen with ^12^C ions against head and neck squamous cell carcinoma (HNSCC) of the oral cavity, larynx, pharynx, and nasopharynx in a fashion that is beneficial for sparing organs at risk such as tissues of the oral cavity, the spinal cord, or bony structures like the mandible and vertebrae has been considered and acted upon. For example, the multi-institutional *in silico* trial designated ROCOCO describes the benefits of particle therapy and in particular CIRT, in a trial of reirradiation for recurrent HNSCC (14). In that study, comparing reductions in mean dose to organs at risk, particle therapy—using protons and carbon ions, both achieved reductions in complications with a dosimetric benefit for carbon ions over protons which they attributed to conformity, that is, dose to the normal tissue as suggested by other studies (15–17).

CIRT facilities have a highly limited capacity to treat the millions of individuals who are diagnosed with cancer each year, and even with new facilities coming online, it remains a limited medical resource where patients should be stratified in order to optimize the use and efficiency of CIRT. Tumors of the head and neck should be ideal for the use of CIRT because head and neck regions have functionally important anatomic sites amenable to dose conformality and the additional cell killing effects of high LET radiations, particularly for low LET-resistant (photon and proton) tumors. Furthermore, identification of patients as potentially radioresistant by omics or other analysis that requires tumor sampling, is less complicated in H&N cancers based upon ease of access to tumor tissue.

Intuitively, DNA repair-related biomarkers would be particularly useful for predicting radiotherapy and chemoradiotherapy outcomes for HNSCC. Ku80, a mediator of DSB repair, was established as the first candidate DNA repair biomarker to show potential predictive value for head and neck radiotherapy in a cohort of archival HNSCC specimens from irradiated patients (18). In this series, Ku80 was overexpressed in half of tumors, and its expression was independent of all clinical and genetic covariates examined. Ku80 overexpression was an independent predictor for both locoregional failure and mortality following radiotherapy (*p* < 0.01) conferring a 9-fold greater risk of mortality at 2 years. Furthermore, using a battery of HNSCC cell lines, tumor growth and metastatic potential were determined in an orthotopic model of oral tongue cancer, including how TP53 mutations influence tumor growth and metastasis (19) and how disruptive mutations in TP53 lead to treatment failure by inhibiting radiation-induced senescence (20). Besides the identification of Ku80 and TP53 as potential negative prognostic indicator in HNSCC, Eschrich et al., using the Radiosensitivity Index (RSI) in a retrospective study of HNSCC treated with radiochemotherapy identified a radiosensitive cohort of patients that saw improved locoregional control (21). However, as argued here, identifying tumors that are likely radioresistant would seem more appropriate for the selection of patients to be treated by CIRT.

Towards that goal of defining radioresistance for patient triage, 38 HNSCC cell lines were collected and interrogated for their γ-ray radioresponse *via* clonogenic survival. An approximately 4-fold range of radiosensitivity as measured by SF2 or SF3.5 was determined. At the molecular level, these 38 cell lines have been assayed for basal gene and miRNA expression as well as DNA methylation, and our future goal is to integrate gene expression, miRNA expression, and methylation patterns with cell survival to characterize radioresponse.

However, for this study, these 38 HNSCC cell lines were agnostically divided into 4 groups, radiosensitive, moderately radiosensitive, moderately radioresistant and radioresistant, based on SF3.5 values. A signature of 37 genes built from the basal gene expression of each cell line was then developed that could stratify these cell lines into their respective radiosensitivity cohorts with 86% accuracy. From these 38 cell lines, five were chosen to characterize the behavior of moderately radioresistant and radioresistant cell lines to ^12^C ion exposures to determine the radioresponse to ^12^C ions, calculate RBE values using different endpoints, and model tumor control probabilities for a series of dose and fraction combinations to expose the variability in tumor control probability when a fixed RBE is used as opposed to a personalized RBE within a radioresistant population of HNSCC tumor cell lines.

## Methods

### HNSCC Cell Culture

HNSCC cell lines were cultured in Dulbecco’s modified essential media (D-MEM) supplemented with 10% Fetal Plus brand fetal bovine serum (FBS, Atlas Biologicals, Fort Collins, CO, USA), penicillin/streptomycin solution (Sigma Aldrich, St. Louis, MO, USA). All cell lines were authenticated by genotyping and validated as negative for mycoplasma contamination by the Molecular Diagnostics Core Services at the Dana Farber Cancer Center, Boston, MA. All cell culture was conducted in incubators at 37°C in ambient 5% CO_2_. See **Table 1** for additional information such as the anatomical site from which the cell line was derived and other information.

**Table 1:** Characteristics of HNSCC cell lines.

Cell line	SF2	SF3.5	P.E.	Anatomical location
584A2	0.45	0.119	0.03	Larynx
CAL-27	0.459	0.248	0.07	Oral cavity
FADU	0.622	0.346	0.44	Hypopharynx
HN30	0.476	0.179	0.38	Pharynx
HN31	0.542	0.265	0.11	LN (HN30)
HN4	0.652	0.307	0.15	REC (larynx)
HN5	0.709	0.414	0.65	REC (oral cavity)
JHU011	0.447	0.19	0.1	REC (larynx)
JHU022	0.442	0.188	0.16	LN (larynx)
JHU029	0.482	0.196	0.25	Larynx
MDA1386LN	0.359	0.117	0.2	LN (MDA1386TU)
MDA1386TU	0.574	0.248	0.08	Hypopharynx
MDA686LN	0.617	0.319	0.02	LN (MDA686TU)
MDA686TU	0.624	0.339	0.08	Oropharynx
MDA886LN	0.342	0.131	0.03	LN (larynx)
OSC19	0.502	0.241	0.03	LN (oral cavity)
PCI13	0.522	0.31	0.03	Oral cavity
PCI-15A	0.342	0.108	0.08	Hypopharynx
PCI-15B	0.392	0.095	0.13	LN (PCI-15A)
PJ34	0.507	0.263	0.14	Oral cavity
SCC15	0.456	0.183	0.07	Oral cavity
SCC25	0.529	0.232	0.09	Oral cavity
SCC4	0.667	0.362	0.24	Oral cavity
SCC61	0.74	0.465	0.64	Oral cavity
SCC9	0.73	0.44	0.28	Oral cavity
Sqccy1	0.688	0.345	0.81	Oral cavity
TR146	0.554	0.256	0.09	REC (oral cavity)
Tul38	0.575	0.332	0.12	Oral cavity
UMSCC1	0.671	0.358	0.58	REC (oral cavity)
UMSCC11A	0.473	0.225	0.02	Larynx
UMSCC14B	0.449	0.152	0.35	REC (UMSCC14A)
UMSCC17A	0.232	0.056	0.14	Larynx
UMSCC17B	0.415	0.168	0.26	E) CT (UMSCC17A)
UMSCC22A	0.473	0.176	0.12	Hypopharynx
UMSCC22B	0.434	0.113	0.07	LN (UMSCC22A)
UMSCC25	0.656	0.372	0.47	LN (larynx)
UMSCC47^a^	0.259	0.075	0.09	Oral cavity
UMSCC4	0.63	0.342	0.2	Oropharynx

a HVP positive. EXT, extension into adjacent tissue; REC, recurrence; LN, lymph node. Anatomical location taken from Zhao et al. (22).

### HNSCC Tumor Gene Expression Microarray Dataset

An expression microarray dataset (GEO accession number GSE 67614) that was generated from 102 tumor samples collected from patients treated with a consistent protocol of surgery followed by radiotherapy based upon a prospective trial that evaluated pathologic risk features, total combined treatment duration, and postoperative radiation therapy (23) was used to evaluate the expression of genes and molecular pathways identified from the cell line gene expression data. The patient pool from which these tumors were isolated were 34% stage III and 54% stage IV, i.e., predominantly high risk, and were divided into cohorts representing those for whom their disease recurred locally/regionally (LR), had distant metastasis (DM), and who showed no evidence of disease (NED). HPV status was not determined directly; however, p16 positivity was seen in samples representing 11 patients and were split 6:5 in the recurrent setting vs. those designated as having no evidence of disease.

### Photon Irradiations

Photon irradiation was conducted at the University of Texas Southwestern Medical Center using a J. L. Shepherd sealed horizontal ^137^Cs-sourced irradiator or at the MD Anderson Cancer Center using the “NASAtron” ^137^Cs irradiator. Dosimetry for these sealed source irradiators was validated on an annual basis. Briefly, for the J. L. Shepherd, irradiator cells in culture were placed on a 360° platform revolving at 13 RPM, irradiated, removed from the irradiator, and immediately returned to the incubator. For the NASAtron irradiations, the source was vertically above the stage and there was no sample rotation. Both devices had dose rates of ~3.25 Gy/min.

### ^12^C Ion Irradiations

All ^12^C irradiations took place at the Centro Nazionale di Adroterapia Oncologica (CNAO) facility in Pavia, Italy. Cells were irradiated in T12.5 cm flasks while immersed in a water bath at 37°C using CNAO’s clinical, therapeutic quality, pencil beam scanning ^12^C-ion beam. A spread-out Bragg peak (SOBP) was created to assure a homogenous ( ± 2.5%) physical dose. The beam quality has been previously characterized (24) and adheres to the recommendations of a NCI special panel on particle beam characterization (25). The dimensions of the SOBP were 17 cm in width, 7 cm in height, and 2 cm in depth. Cells were centered in the SOBP within a leucite holder with cells set back-to-back such that the depth of the cells in the upstream flask was 80 mm water equivalent depth (WED) while the position of cells in the downstream flask was 84 mm of WED. LETs at the positions where the cells were aligned were 74.1 and 89.3 keV/µm, respectively. (No difference in biological response was seen based upon position.) The entrance LET was 16.4 keV/µm at a depth of 0.15 mm. The physical dose rate was typically 0.60 Gy/min.

### Irradiation of Cells With a Carbon Ion SOBP

To ensure a consistent SOBP, cells were suspended in a circulating water phantom maintained at 37°C in sealed T-12.5 flasks filled to the neck with complete cell culture medium containing 2% FBS for a roughly 5-min pre-exposure. This configuration provided a complete liquid/plastic interface with no ion deflection due to the presence of air. Immediately after irradiation, 2% FBS-containing media was aspirated and replaced with 5 ml of complete media containing 10% FBS for incubation.

### Clonogenic Cell Survival Assays

Cells undergoing log phase growth at roughly 70%–80% maximum cell culture density were trypsinized and then seeded into T-12.5 flasks at low density in complete growth medium 8 h prior to irradiation. Five minutes prior to irradiation with either γ-rays or carbon ions, cell culture flasks were filled to the neck with complete growth media containing 2% FBS. Cells were irradiated with doses of 1, 2, 4, 6, and 8 Gy of γ-rays, or 0.5, 1, 2, 4, and 6 Gy carbon ions. Following irradiation, growth medium containing 2% FBS was immediately aspirated and replaced with growth medium containing 10% FBS and dishes were allowed to incubate for ~10 population doublings based on cell-specific doubling times. The use of media with 2% FBS was simply to limit the overall volume of FBS that would be used if media containing 10% FBS was used to completely fill the T12.5 flasks for only a few minutes as was necessary at CNAO. (See above.) Using 2% FBs for such a limited time had no effect on cell growth or radioresponse.

Following incubation, cultures were rinsed with phosphate-buffered saline at a pH of 7.4 and fixed in a solution of 0.5% crystal violet and 10% methanol in water. After staining and drying, colonies were counted to determine the number of surviving cells following irradiation. Only colonies identified as having more than 50 cells per colony were scored as surviving, and the surviving fraction was determined by dividing the number of colonies by the product of the plating efficiency of the cell line multiplied by the number of cells seeded.

### Survival Curve Fits

Survival curves were fitted based upon the Repairable Conditionally Repairable (RCR) Model as described in Equation 1 where *d* is the dose per fraction and *a*, *b*, and *c* are parameters determined using a curve fitting algorithm (26).


(1) $$S(d)=e^{-ad}+bde^{-cd}$$

The γ-ray survival assays were performed at least twice for each cell line. If the coefficient of variation at 2 Gy was greater than 25%, they were repeated.

### RBE Calculations

RBE values were calculated by comparing a radiosensitivity value from ^137^Cs exposures (reference) to that same radiosensitivity value determined from ^12^C exposures (test) as in **Equation 2**.


(2) $$\text{RBE}=\frac{\text{Dose,reference}}{\text{Dose,test}}$$

Radiosensitivity parameters included:

#### Dose at 10% Survival

The dose at SF_10%_ was calculated using values generated with the RCR model as described in Equation 1.

#### ${\bar{D}}_{\text{parm}}$

$\bar{D}$ was calculated using the parameters of the RCR model as shown in Equation 3 (26).


(3) $$\bar{D}=\frac{1}{a}+\frac{b}{c^{2}}$$

#### ${\bar{D}}_{\text{AUC}}$

Here, $\bar{D}$ is calculated using a trapezoidal method of the area under the curve (AUC) of the survival curve assay. The trapezoidal method is similar to a Reimann sum with the exception that the area is approximated using trapezoids as opposed to rectangles.

#### Limiting slope, *D*_0_

The limiting slope, *D*_0_, represents the linear portion of the RCR fit located at the distal end of the survival curve. *D*_0_ was calculated by plotting the linear portion of the RCR line of best fit in MATLAB and using a linear to calculate the slope. The relationship between the slope of the linear portion and *D*_0_ is given in Equation 4.


(4) $$D_{0}=\frac{-1}{\text{slope}}$$

### Transcriptomic Analysis of HNSCC Cell Lines and Tissues

#### Labeling and Hybridization of Microarrays

The tumor data set was generated using Illumina Whole Genome HumanWG6 v2 arrays (GEO accession number GSE67614). The v3 Illumina Expression BeadChip was used to generate transcriptome profiles for the HNSCC cell lines. Each RNA sample with 0.5 µg of total RNA was amplified using the Illumina TotalPrep RNA amplification kit with biotin UTP (Enzo) labeling. The Illumina TotalPrep RNA amplification kit uses T7 oligo(dT) primer to generate single-stranded cDNA followed by a second-strand synthesis to generate double-stranded cDNA which is then column purified. *In vitro* transcription was done to synthesize biotin-labeled cRNA using T7 RNA polymerase. The cRNA was column purified and then checked for size and yield using the Bio-Rad Experion system. A total of 1.5 µg of cRNA was hybridized for each array using the standard Illumina protocols with streptavidin-Cy3 (Amersham, Amersham, UK) being used for detection. Slides were scanned on an Illumina Beadstation. Summarized expression values for each probe sets were generated using BeadStudio 3.1 (Illumina Inc., San Diego, CA, USA).

#### Preprocessing and Data Analysis for Gene Expression Profiling

The Illumina BeadChip expression data were background subtracted and quantile–quantile normalized across samples using the MBCB algorithm (27–29). Normalized gene expression values were used for all the subsequent analysis. The clustering analysis was performed by calculating Euclidean distances and clustered by average method using the hclust function from the R base package.

### Classification of Radiosensitivity Groups in HNSCC Cell Lines Using Gene Expression Profiles

The gene expression values from 38 HNSCC cell lines were ranked by *p*-values generated from an *F*-test using the R limma package. The top-ranked genes corresponded to those that significantly changed in more than one of the radiosensitivity groups. Feature selection was performed by incrementally expanding the gene list from 4 to 500 from the top of the gene ranking. Classification models were built using a support vector machine (SVM) algorithm and the models were validated using a 10-fold repeated cross-validation.

#### Molecular Pathway Analysis

Functional pathway analysis was performed using the Gene Set Enrichment Analysis (GSEA 4.0.1) and Ingenuity Pathways Analysis (IPA) online software packages. Genes were ranked by *p*-values calculated from moderated *t*-test using the R limma package. The R limma analysis was performed to compare resistant cell lines vs. other cell lines or tumors where there was local recurrence (LR) vs. tumors where there was NED in patients treated with postoperative radiotherapy. A false-discovery rate (FDR) <0.4 was used as a cutoff for significantly enriched pathways and then interrogated against the GSEA Hallmark Pathways by Leading Edge Analysis for pathway enrichment using the metrics of *p*-value, FDR, and Normalized Enrichment Scores, whereas the IPA analysis focused on pathways were enriched based upon a Fisher’s test with a *p*-value of 0.05 used as a cutoff. *Z*-scores for these pathways were then calculated with negative scores representing downregulation and positive scores representing upregulation of a given pathway.

### Tumor Control Probability

The calculation of tumor control probability (TCP) is adapted from Antonovic et al. (30) and is summarized in **Equation 5** where *N*_vox_ is the number of voxels in an *in silico* tumor, *N_i_* is the number of cells in voxel *i*, and *S_i,j_ (d,L,pO2)* is the surviving fraction in voxel *i* at fraction *j* with dose *d*, oxygen partial pressure *pO_2_*, and LET *L*.


(5) $$\text{TCP}=\exp\left\{-\sum_{i=1}^{N_{\text{vox}}}N_{i}\prod_{j=1}^{n}S_{i,j}(d,L,pO_{2})\right\}$$

A spherical tumor with a radius of 0.6203 cm (total volume 1 cm^3^) was assumed to contain 10^8^ cells distributed equally across the voxels in the *in silico* tumor simulation. The survival model utilized is the Repairable Conditionally Repairable (RCR) model which can account for changes in survival due to dose, LET, and partial oxygenation, as shown in **Equation 6**.


(6) $$S(d,L,pO_{2})=e^{-a(L)d/\tilde{O}(L,pO_{2})}+b(L)d/\tilde{O}(L,pO_{2})e^{-c(L)d/\tilde{O}(L,pO_{2})}$$

As only the effect of dose on survival was accounted for **Equation 5** was simplified to **Equation 7** and **Equation 6** was simplified to **Equation 1**.


(7) $$\text{TCP}=\exp\left\{-\sum_{i=1}^{N_{\text{vox}}}N_{i}\prod_{j=1}^{n}S_{i,j}(d)\right\}$$

To take into account the effect of dose per fraction and the number of fractions on survival, **Equation 1** was modified to **Equation 8** where *d* is the dose per fraction and *n* is the number of fractions. In each simulation, the dose per fraction, *d*, is kept constant and the number of fractions, *n*, is varied to generate a TCP curve.


(8) $$S(d)={\left(e^{ad}+bde^{-cd}\right)}^{n}$$

## Results

**Figure 1:**
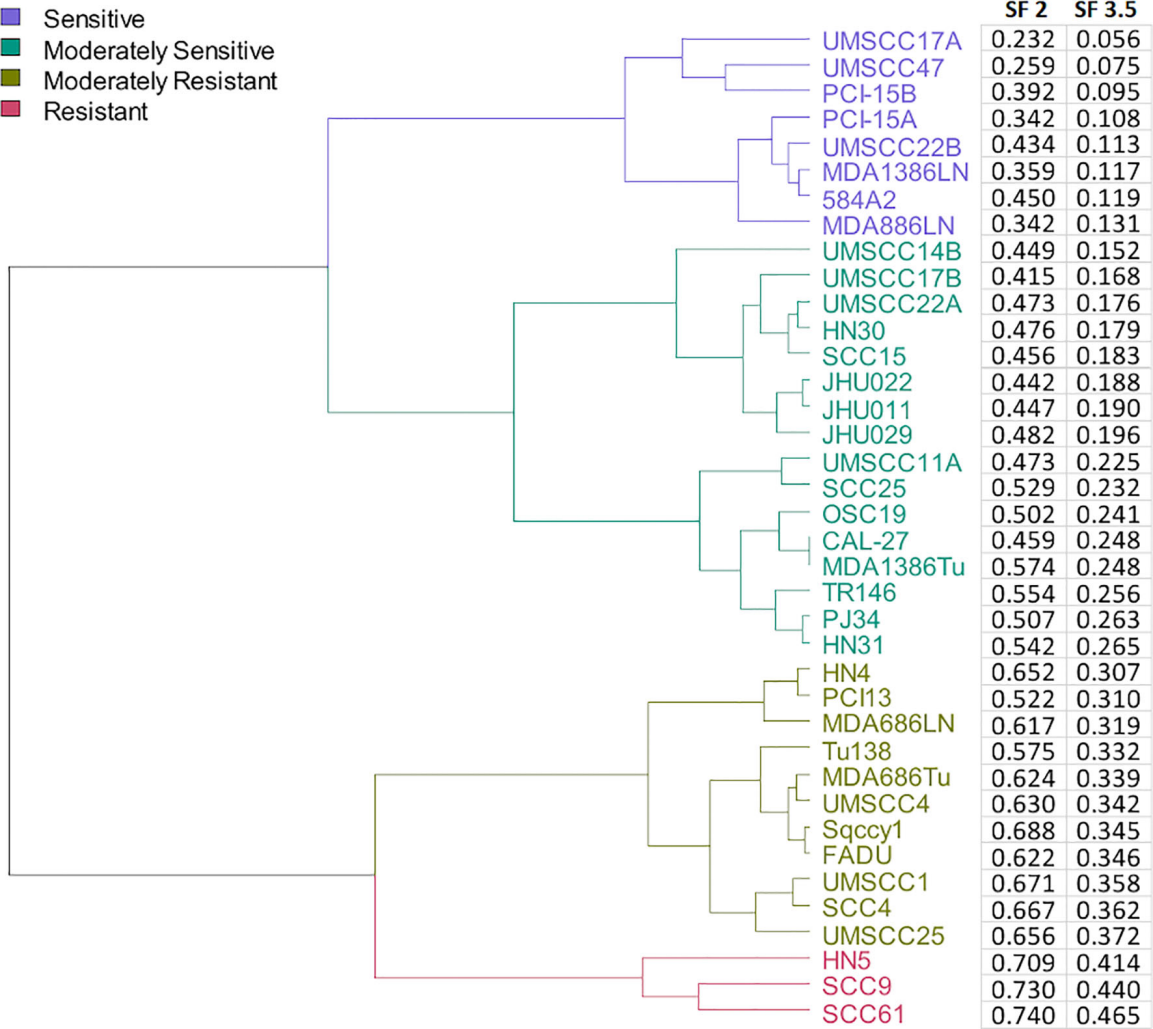
Dendrogram of cell lines clustered by their radiosensitivity at 3.5 Gy and agnostically grouped into 4 clusters based upon radiosensitivity.

A collection of 38 HNSCC cell lines was tested for radioresponse *via* clonogenic cell survival. Surviving fraction at 2 and 3.5 Gy (SF2, SF3.5, respectively) was determined based upon the fitted values for SF2 and SF3.5 from the survival curves. The cell lines were clustered into four groups based upon Euclidean distances using the SF3.5 values for each cell line as depicted in **Figure 1**. Those groups are described as sensitive (S), moderately sensitive (MS), moderately resistant (MR), and resistant (R). The cluster is accompanied by the cell line identity and the respective SF3.5 values. SF3.5 was chosen based upon the conclusions of Johansen et al. (31), who suggest SF3.5 as more representative of radiosensitivity where a larger dose per fraction might be used as in stereotactic ablative radiotherapy (SAbR) or CIRT.

**Figure 2:**
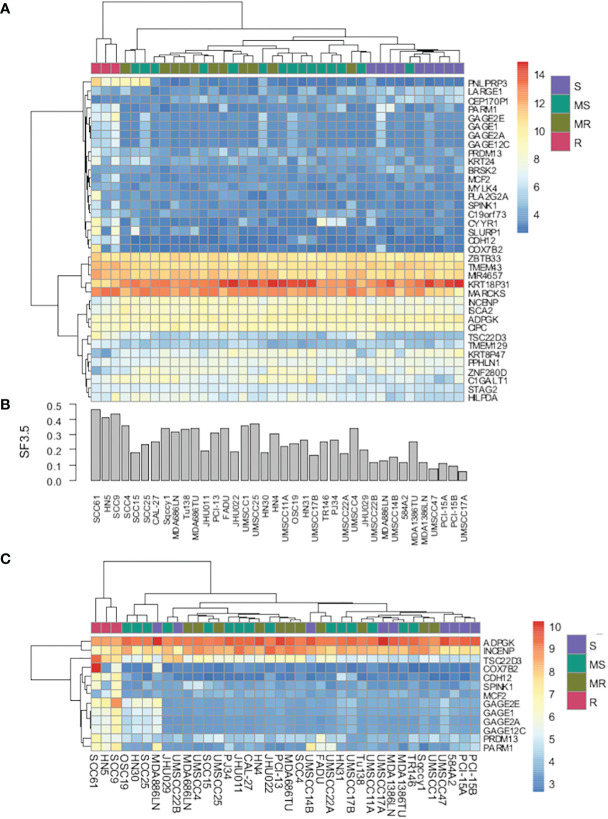
**(A)** Heat map of gene expression using a 37-gene signature that segregates cell lines by radiosensitivity cluster. **(B)** Bar chart of radiosensitivity (SF3.5) for each cell line. **(C)** Heat map of gene expression using a 13-gene signature that segregates radioresistant cells from all other cell lines.

To determine whether there were differences in gene expression underlying these radiosensitivity cohorts, an SVM model was trained using the most significantly changed genes. As depicted in **Figure 2A**, a 37-gene panel could place the cell lines within their respective radiosensitivity cohort with an accuracy of 86%. **Figure 2B** represents the SF3.5 for the cell lines directly above in **Figure 2A** and reflects the trend in cell line radioresponse from radioresistant on the left to radiosensitive on the right. Furthermore, if cell line segregation was adjusted to identify the three radioresistant lines from all others, only 13 genes were required (**Figure 2C**). Genes segregating the R cohort include those associated with radio/chemoresistance (GAGE12C, GAGE2E, SPINK1), metabolic processes (PNLIPRP3), proliferation migration, invasion and metastasis (PARM1, CDH12, CYYR1, GAGE12C), and inhibition of apoptosis (SPINK1).

**Figure 3:**
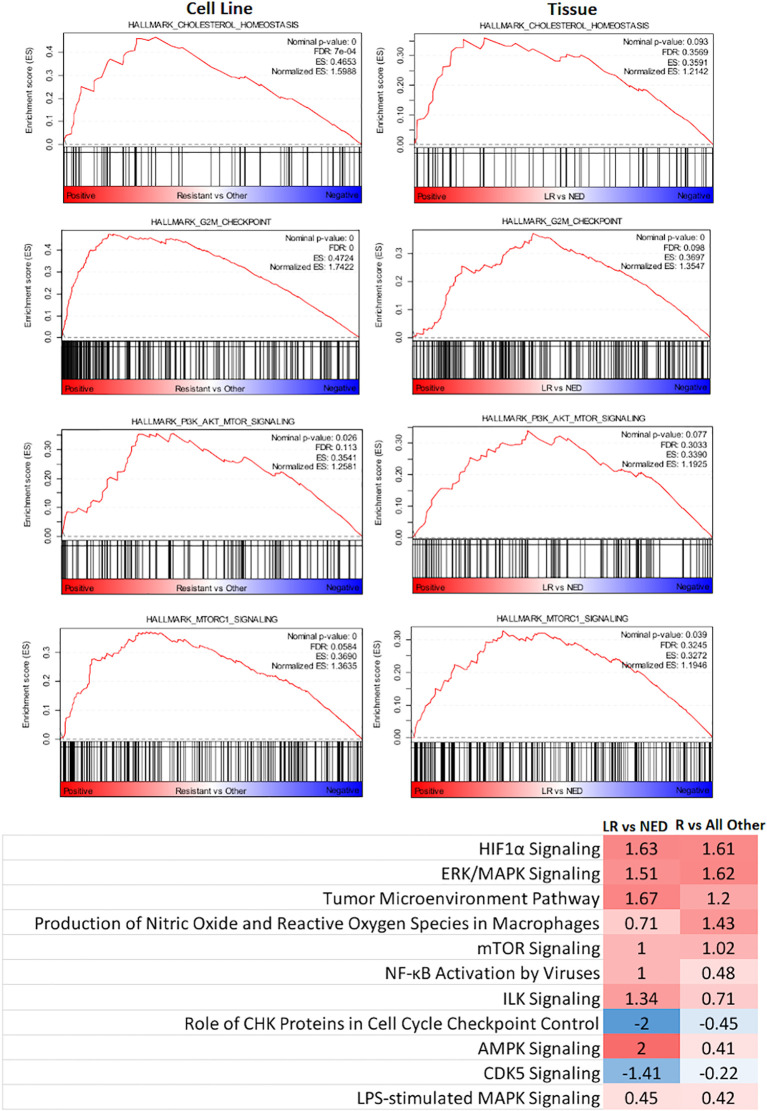
Identification of key signaling pathways that segregate radioresistant cells from others and comparison with key signaling pathways identified in HNSCC tumors treated by PORT and classified by their treatment response: no evidence of disease (NED) and local recurrence (LR).

GSEA and IPA were used to examine differences in Hallmark Pathways (GSEA) and canonical pathway signaling (IPA). As shown in **Figure 3**, when the R cohort was compared with all others, cholesterol metabolism, G2M checkpoint, PI3K/AKT/MTOR, and MTORC1 pathways were enriched with Normalized Enrichment Scores being 1.5988, 1.7422, 1.258, and 1.363, respectively. IPA pathway analysis identified HIF1a and ERK/MAPK pathways as upregulated along with a number of other pathways that were up- or downregulated based upon *Z*-scores.

These same pathways were then interrogated in a gene expression dataset derived from 102 flash-frozen HNSCC tumor specimens from patients treated by postoperative radiotherapy (PORT) using a consistent treatment protocol (23, 32, 33). The gene expression data from 49 patients characterized as having NED and 35 patients characterized as having had LR were used. As shown in **Figure 3**, the GSEA Hallmark Pathway cholesterol metabolism, G2M checkpoint, PI3K/AKT/MTOR, and MTORC1 were also enriched in the LR group when compared with those designated NED. Not surprisingly, their Normalized Enrichment Scores were somewhat lower than that seen in the cell lines.

**Figure 4:**
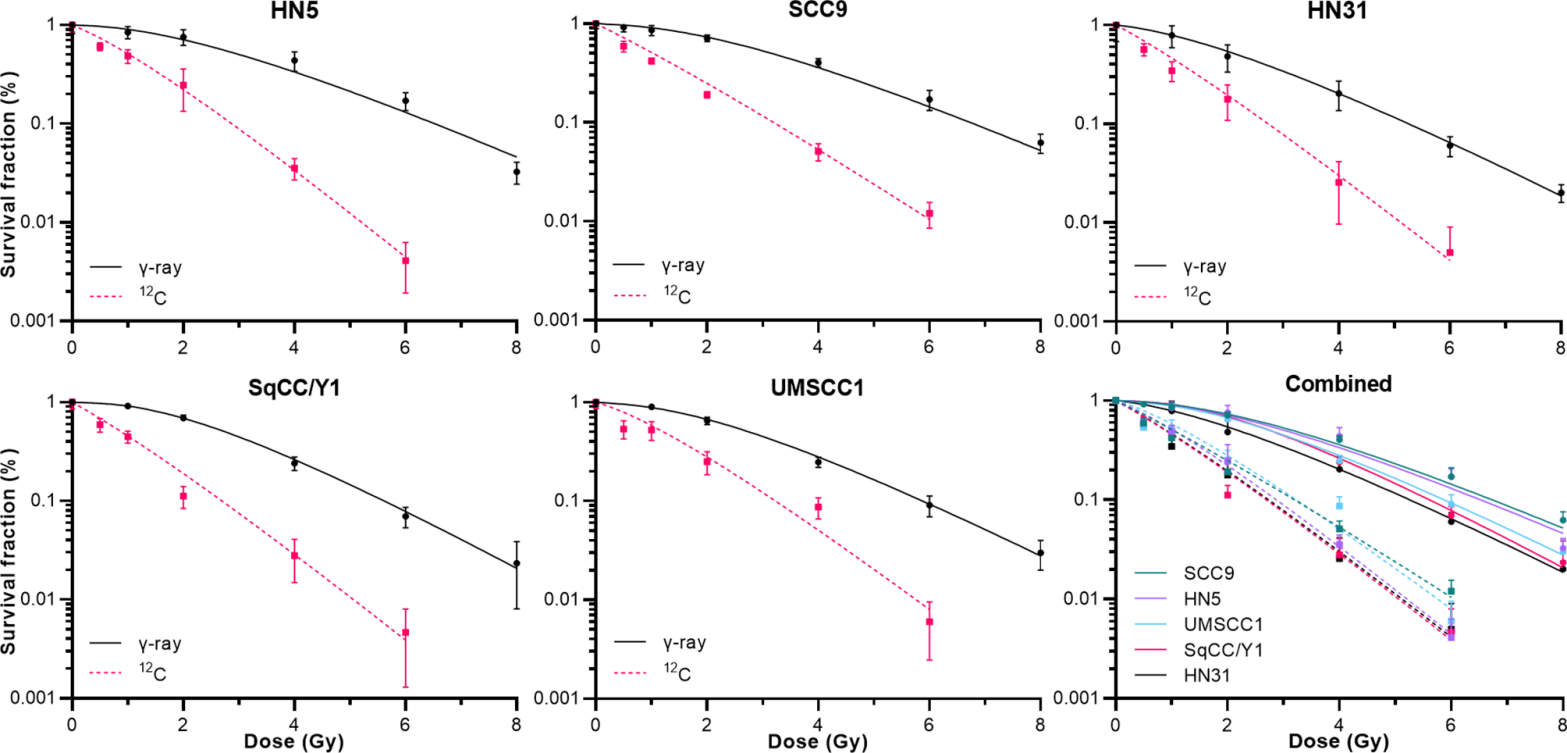
Clonogenic cell survival for the 5 cell lines described in **Table 2** when irradiated by either γ-rays or ^12^C at 400 Mev/u. The lower right panel shows all data combined.

Next, we examined the ^12^C radioresponse of five HNSCC cell lines whose SF3.5 ranged from average to resistant when compared with the larger panel of 38 cell lines to determine if ^12^C irradiation can overcome radioresistance. Radiation survival curves were generated using ^137^Cs as a low LET radiation and ^12^C ions were generated by the Carbon Therapy Center in Pavia, Italy (CNAO). The cell lines SqCC/Y1 and UMSCC1 were chosen to represent the range of SF3.5 in the MR group with an emphasis on the high end of response; HN31 represents the highest SF3.5 in the MS cohort; and HN5 and SCC9 represent the R cohort. Survival curves for these cell lines are seen in **Figure 4**. The RBE for ^12^C ions was calculated at: 10% survival; by mean inactivation dose $\left(\bar{D}\right)$ using the parameters of the repair-conditionally repairable curve fitting algorithm (26); $\bar{D}$ using a Reimann sum approach; and the limiting slopes (*D*_0_) for each cell line. Those values are found in **Table 2**.

**Table 2:** Limiting slopes, mean inactivation doses, and RBEs using different methods of determination.

Cell line	RBE_SF10%_	$\text{RBE}{\bar{\text{D}}}_{\text{parm}}$	$\text{RBE}{\bar{\text{D}}}_{\text{AUC}}$	RBE_D0_
**SCC9**	2.11	2.55	2.55	1.51
**HN5**	2.27	2.61	2.56	1.93
**UMSCC1**	1.83	2.09	2.07	1.50
**SqCC/Y1**	2.08	2.58	2.57	1.51
**HN31**	1.92	2.14	2.12	1.61
**Average**	2.04	2.39	2.37	1.61
**Std dev**	0.17	0.26	0.25	0.18
**CV**	0.08	0.11	0.11	0.11

RBE_SF10%_, RBE calculated using 10% survival; $RBE{\bar{D}}_{parm}$ , RBE calculated using mean inactivation dose derived from RCR parameters; $RBE{\bar{D}}_{AUC}$ , RBE calculated using mean inactivation dose derived from Reimann sum; RBE_D0_, RBE calculated as ratio of limiting slopes.

**Figure 5:**
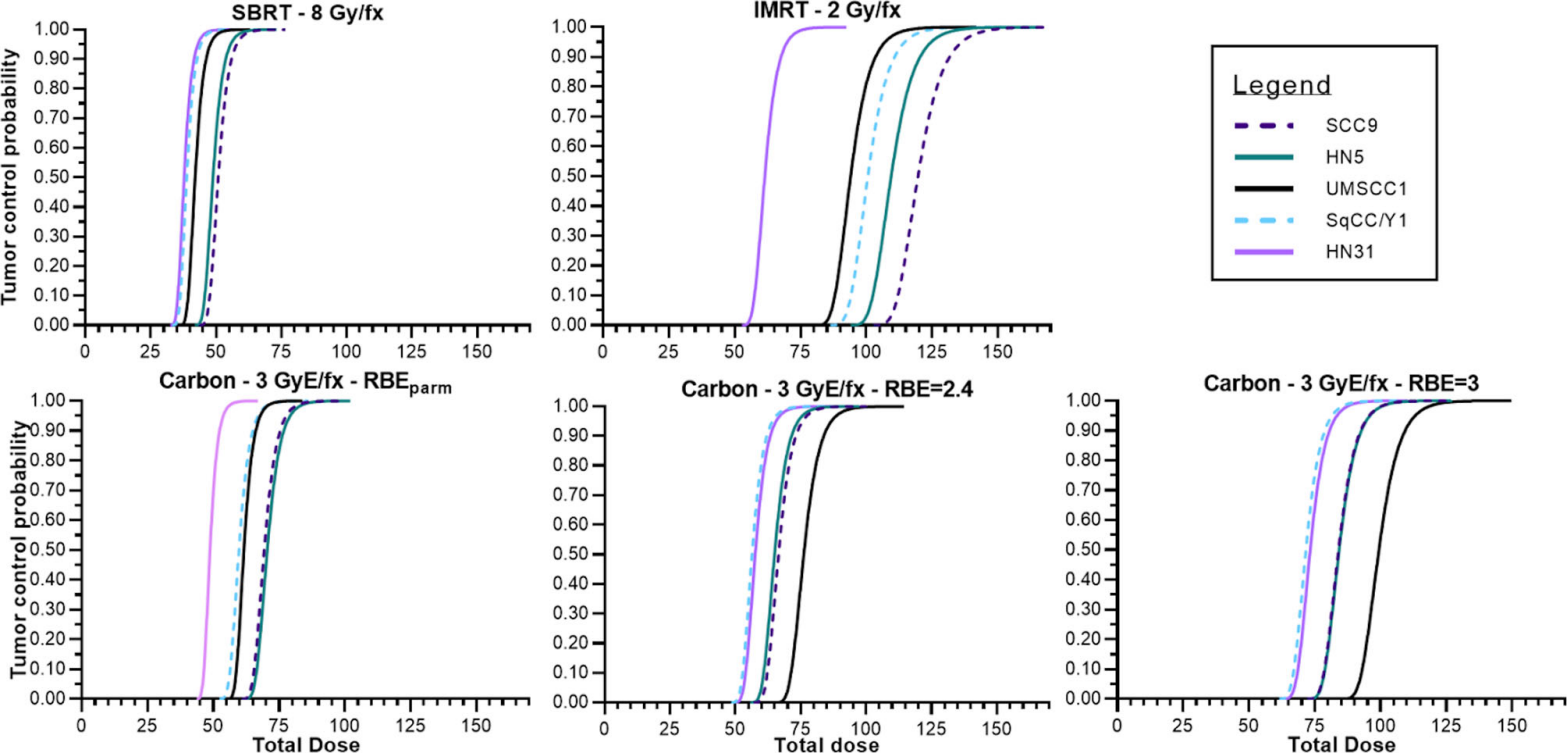
Tumor control probabilities for various treatment modalities across the panel of HNSCC cell lines assuming no hypoxia, tumors that are a volume of 1 cm and containing 10^8^ tumor cells. Photon IMRT, SAbR, ^12^C using a fixed RBE to determine GyE, ^12^C using the RBE associated with the cell line used for tumor modeling.

Using the survival data for both γ-rays and clinical ^12^C ions from the five HNSCC cell lines, TCP curves were generated based upon Antonovic etal. (30). The calculation of TCP differs slightly from the study of Antonovic et al. (30) in that the tumor model created was fully oxygenated and the LET is fixed. Fractionation schedules (IMRT, ^12^C) were based upon schedules used in recurrent H&N cancers treated at the Shanghai and Heidelberg Heavy Ion radiotherapy facilities (34, 35) where we chose an RBE value of 3, which is in line with that used clinically, as well as the RBE values determined for each cell line. These TCP curves are depicted in **Figure 5**.

**Figure 6:**
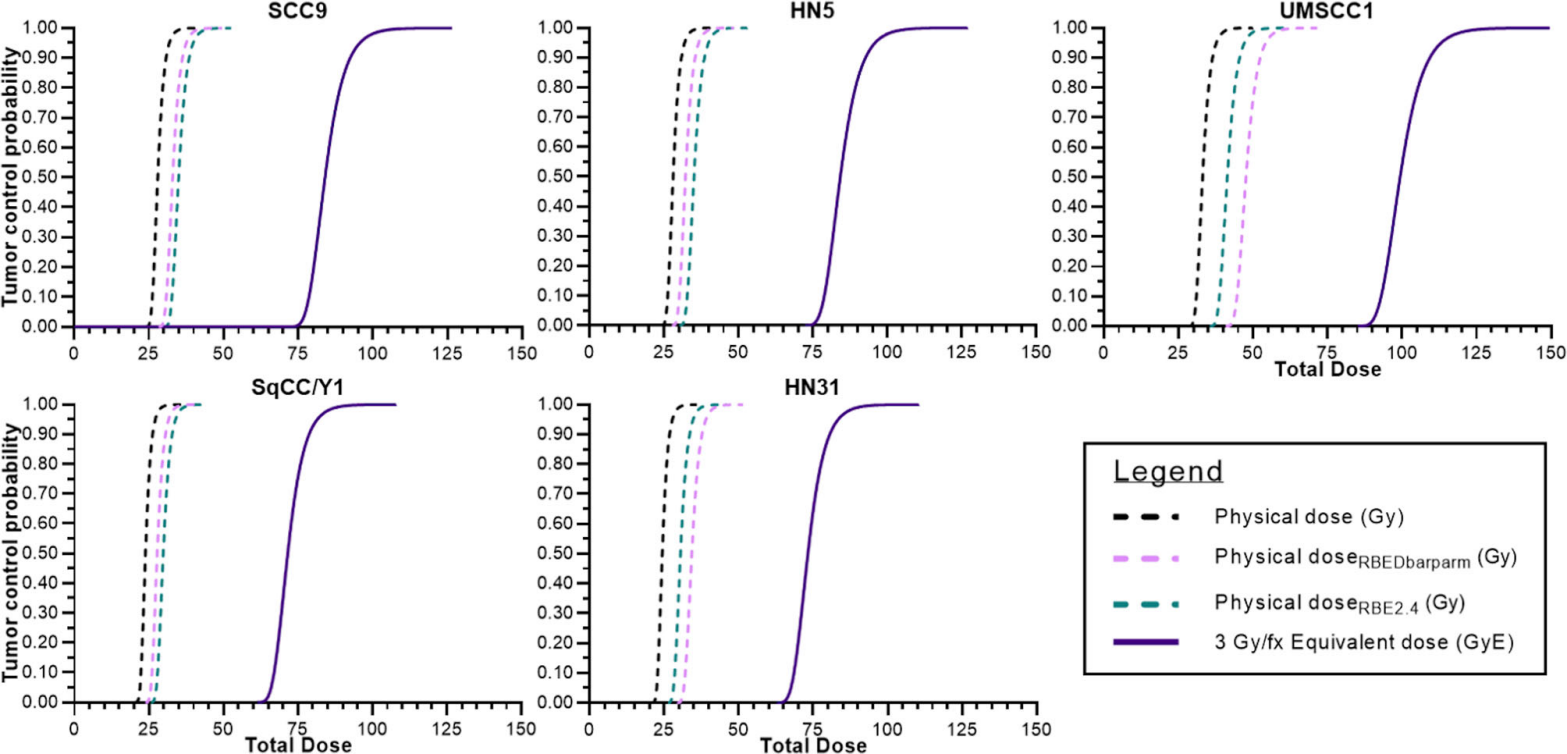
Tumor control probabilities for each cell line after ^12^C ion radiotherapy: left line is physical dose; far right line represents TCP when a clinical dose equivalent (GyE) based upon a fixed RBE is used; and the intermediate dashed line is the physical dose necessary to achieve the same TCP as the far right curve. The interval between the two physical doses is the underdosing for each tumor. That value at a TCP of 70% is seen in **Table 3**.

Lastly, in **Figure 6**, TCP curves representative of each cell line using the physical dose (solid line) and the GyE dose (red dashed line) are plotted, which represent the expected TCP using a generic RBE of 3. The blue dashed line in each curve represents the physical dose necessary to accomplish the same TCP as the generic RBE of 3. The differences in physical dose would represent the “underdosing” of radioresistant tumors and are found in **Table 3**.

**Table 3:** Comparisons of GyE based upon use of a fixed RBE vs. the actual RBE for a given cell line and the dose differential for a tumor control probability of 70%.

Cell line	Dose (GyE) if RBE = 3	Physical dose (Gy)	Dose (GyE) if actual RBE used	RBE (actual)	Difference in total GyE (fixed RBE vs. actual)
**SCC9**	86.9	28.9	73.9	2.55	13
**HN5**	87.2	29	75.9	2.61	11.3
**UMSCC1**	102.8	34.2	71.5	2.09	31.3
**SqCC/Y1**	74.1	24.7	63.7	2.58	10.4
**HN31**	75.8	25.2	54	2.14	21.8

## Discussion

HNSCC is the sixth most prevalent cancer in the world, and despite many treatment options, local or regional recurrence is still as high as 30%–50% after surgery or surgery plus radiochemotherapy. The alternatives for recurrent HNSCC include surgery with or without adjuvant radiochemotherapy with survival rates of 40%–66% (36), but the rates of grade 3 or grade 4 toxicities can be as high as 40% (37–41) with only 1 in 3 patients surviving reirradiation without recurrence or severe complications (42).

The high total doses from prior radiotherapy limit the total dose acceptable for reirradiation based upon the toxicity to organs at risk. However, a retrospective analysis of patients treated at the Heidelberg Ion-beam Therapy Center (HIT) by reirradiation for recurrent HNSCC with CIRT developed little in the way of either acute or late severe (>Grade III) toxicity (35) using a median total dose of 51 GyE in 3 GyE fractions. The Shanghai Proton and Heavy Ion Center in a trial of recurrent HNSCC found similar results (34). Late toxicities (> Grade III) were seen in 7.1% of patients as compared to HIT where late toxicities occurred in 14.5% of patients. The primary tumor sites in these two series of patients were vastly different with Shanghai for instance having ~83% of cases represented by squamous carcinomas (nasopharyngeal cancer representative of 78% of all cases) while only 28% of the HIT cohort was squamous carcinoma. These differences in patient population likely drive the differences in toxicity profiles and compare well to reirradiation trials for head and neck cancers using proton therapy where late toxicities ranged from 7% to 8%, 20%, and 24.6%, respectively (43–45).

The comparable overall improvements in late normal tissue toxicities in H&N cancers treated with CIRT suggest the potential for overall improvements in outcome in primary and recurrent disease as well as the potential for dose escalation in tumors identified as radioresistant. Also, given the limitations of access, if patients with tumors that are radioresistant can be identified, the argument could be made to treat those patients where possible with ^12^C ions. Radioresistant HNSCC can be manifested *via* a number of mechanisms, the predominant mechanism being hypoxia, but resistance may also be through acquired mechanisms of enhanced DNA repair, abrogation of apoptosis or through the development of a tolerogenic immune environment. ^12^C ions—or for that matter other ions separately or in combination—should at least partially overcome these limitations.

Determining radioresistance in a clinically useful manner is not straightforward as there are currently no clinically useful biomarkers of radioresistance in HNSCC with the possible exception of HPV status. Because of the ease with which tumors can be biopsied in H&N cancers, an omics approach is feasible. To test this approach, 38 HNSCC tumor cell lines were tested for radiosensitivity *via* clonogenic survival. Basal gene expression analysis was then tested as a surrogate for clonogenic survival and found to be capable of segregating the most radioresistant cell lines from the other cell lines. Furthermore, the gene expression patterns in these radioresistant cell lines supported their classification as radioresistant. Besides identifying some of the more commonly identified genes and pathways associated with radioresistance, one of the more intriguing finds is the increased expression of the GAGE genes. These genes are not expressed in normal tissue with the exception of testes. In tumors, members of this family (GAGE1 and 2) are CD4+T cell antigens, attracting T cells into tumors. GAGE12 family members have been shown to increase both radio- and chemoresistance and metastasis (46–48). Other genes identified included SPINK1, an inhibitor of apoptosis that has been associated with chemoresistance (49, 50), and PARM1 which is an androgen-related gene that drives tumor proliferation (51, 52). Mechanistic analysis of any role for the genes in HNSCC radioresistance is warranted.

Assuming that one will be able to identify radioresistance so that patients could be triaged to receive ^12^C ion therapy is of little benefit if this cohort of patients is then not treated to full potency. That potency is based upon an understanding of the dose equivalence for a given situation—or individual. Unlike stochastic processes such as radiation-induced carcinogenesis which uses the Seivert (Sv) for dose equivalent, dose equivalent for ^12^C radiotherapy is described as GyEq, GyE, or GyRBE because there is no unit definition for dose equivalent in a deterministic setting. GyEq relies on the determination of the RBE for a given endpoint. The earliest determinations of RBE, still used today, were based upon cell survival in mostly rodent (CHO) cell lines and the human radioresistant salivary tumor cell line HSG, which was determined to be contaminated with HeLa cells (53, 54). The initial ^12^C scattered beam at NIRS was normalized to the dose-averaged LET at 80 KeV/µm using their clinical neutron RBE of 3.0 as they were equally effective (55). This along with their experience with neutron exposures led them to use this value in their NIRS treatment planning system across all tumor sites. However, it is likely that “clinical RBE” is not a fixed value and should be personalized where possible. Furthermore, RBE can be a poor descriptor of the radiobiology associated with charged particle therapy as there are many factors that determine a RBE value, and it suffers from the inability to make direct comparisons from one method of determination to another or treatment regime to another (26, 30, 56). Therefore, we determined the ^12^C RBE in a small cohort of moderate to radioresistant cell lines using multiple endpoints and then applied the survival and RBE values to model tumor control probability and asked whether individualization of RBE could be consequential when compared to a generic value.

**Figure 5** depicts tumor control probability curves for the 5 tumor cell lines. Dose fractionation schemes include a standard 2 Gy/fraction scheme, a SAbR fractionation scheme of 8 Gy per fraction, and a 3 GyE per fraction approach (generic RBE of 3). As expected, TCP varied according to the radiosensitivity of the tumor with the most resistant tumors requiring greater overall total doses to achieve the modeled tumor cure. The TCP curves for the HN31, which is intermediate in radiosensitivity, is far to the left of the more radioresistant lines in the upper left panel (IMRT 2 Gy/fraction) and at 60–70 Gy total dose TCP is roughly between 30% and 90%. The remaining TCP curves for the more radioresistant cells falls well outside with total doses of 90–120 Gy required to achieve 50% control. Total doses for the TCP of a SAbR regimen are far less but also suffer from a lack of efficacy for very radioresistant tumors and would be expected to be constrained by an increased risk for normal tissue complications. The TCP curves for ^12^C, because this is GyE and uses a more conventional fraction size, is comparable with the conventional 2 Gy/fraction regimen. The SabR approach with ^12^C ions may be more effective for carbon ions but suffers the same complications as a conventional SAbR approach within the radioresistant subset.

In **Figure 6**, we attempted to determine the underdosing for a given tumor if a fixed RBE vs. an individually determined RBE was used. TCP curves include (a) the TCP using the generic RBE of 3 for each cell line from **Figure 5**; (b) the 12C physical dose associated with the TCP determined using the generic RBE; and (c) the physical dose if the TCP curve based upon an RBE of 3 were modified by the RBE determined for each cell line. That difference in the physical dose curves could be considered as an “underdosing” of ^12^C physical dose and it is consequential in that it reflects the need to understand the intrinsic radioresistance for a given tumor. Those values of “underdosing” at a TCP of 70% are given in **Table 3**.

In this manuscript, we have attempted to draw attention to the need for individualizing therapy based upon the intrinsic radioresponse of a tumor. Head and neck squamous cells were used because of the recent trials where HNSCC are being treated with ^12^C and because HNSCC outcomes have seen little improvement. At least for cell lines, gene expression was able to segregate radioresistant tumor cell lines from cell lines less resistant. One could argue that 38 cell lines do not bring enough diversity of radiosensitivity as the most radioresistant cell lines were few in number. Also, while there was overlap in the enrichment of specific pathways in common between cell lines and tumors that were treated with PORT, it is evident that much work is needed, irrespective of the gene expression analysis.

Using five cell lines whose radiosensitivity ranged from moderate to resistant across the 38 cell line panel, the potential impact of intrinsic radiosensitivity was tested by determining TCP curves using various radiation regimens for both γ-ray and ^12^C exposures. This exercise highlighted the potential for underdosing radioresistant tumors when generic rather than personalized RBEs were used, which would negate the impact of triaging patients with radioresistant tumors.

We understand that our TCP calculations reflect fully oxygenated tumors, did not reflect tumor response to varied LETs, etc., but our goal is more one of relative comparisons and not necessarily quantitative comparisons. What we require are data from actual tumors treated with curative intent using X-rays and ^12^C ions with different dose and fractionation schedules and a realistic understanding of the impact of tumor hypoxia to challenge and ultimately improve upon the biophysical modeling of CIRT with the intent to ultimately personalize therapy.

## Data Availability Statement

The original contributions presented in the study are included in the article/supplementary materials. Further inquiries can be directed to the corresponding author.

## Author Contributions

MS conceived of and carried out experiments, wrote the manuscript, and led the research program. AD conceived of and carried out experiments, edited the manuscript, and participated intellectually. AP was responsible for radiation physics, helped develop the beam configuration, and wrote a portion of the manuscript. DS was partly responsible for designing and carrying out experiments. MC was responsible for 12C dosimetry, developing the SOBP for CNAO, and all physics aspects of 12C irradiation. AF was the CNAO biology contact person. She helped facilitate experiments and helped with the design of our irradiation setup at CNAO. JY was responsible for collecting the panel of H&N tumor cell lines, designed and carried out experiments to determine the X-ray survival parameters for these cells, and provided DNA and RNA for omics analysis. EP carried out additional survival X-ray survival curves, plotted all data, and calculated survival fits and tumor control probability curves. BS conceived of and carried out both X-ray and 12C experiments. He was involved in the interpretation of data and wrote sections of this manuscript. LD was responsible for all omics analysis, interpretation of that data, and wrote sections of the manuscript. All authors listed have made a substantial, direct, and intellectual contribution to the work and approved it for publication.

## Funding

Funds were provided through the Department of Radiation Oncology Seed Grant Program to MS as well as the David A. Pistenmaa M.D., Ph.D. Distinguished Chair in Radiation Oncology to MS. The funders were not involved in the study design, collection, analysis, interpretation of data, the writing of this article or the decision to submit it for publication.

## Conflict of Interest

The authors declare that the research was conducted in the absence of any commercial or financial relationships that could be construed as a potential conflict of interest.

## Publisher’s Note

All claims expressed in this article are solely those of the authors and do not necessarily represent those of their affiliated organizations, or those of the publisher, the editors and the reviewers. Any product that may be evaluated in this article, or claim that may be made by its manufacturer, is not guaranteed or endorsed by the publisher.
